# Registration error of the liver CT using deformable image registration of MIM Maestro and Velocity AI

**DOI:** 10.1186/s12880-017-0202-z

**Published:** 2017-05-04

**Authors:** Nobuyoshi Fukumitsu, Kazunori Nitta, Toshiyuki Terunuma, Toshiyuki Okumura, Haruko Numajiri, Yoshiko Oshiro, Kayoko Ohnishi, Masashi Mizumoto, Teruhito Aihara, Hitoshi Ishikawa, Koji Tsuboi, Hideyuki Sakurai

**Affiliations:** 1 Proton Medical Research Center, University of Tsukuba, 1-1-1, Tennoudai, Tsukuba, Japan; 2 Division of Radiology, Ibaraki Prefectural Central Hospital, 6528, Koibuchi, Kasama, Japan

**Keywords:** Deformable image registration, Rigid image registration, Liver, Proton beam therapy

## Abstract

**Background:**

Understanding the irradiated area and dose correctly is important for the reirradiation of organs that deform after irradiation, such as the liver. We investigated the spatial registration error using the deformable image registration (DIR) software products MIM Maestro (MIM) and Velocity AI (Velocity).

**Methods:**

Image registration of pretreatment computed tomography (CT) and posttreatment CT was performed in 24 patients with liver tumors. All the patients received proton beam therapy, and the follow-up period was 4–14 (median: 10) months. We performed DIR of the pretreatment CT and compared it with that of the posttreatment CT by calculating the dislocation of metallic markers (implanted close to the tumors).

**Results:**

The fiducial registration error was comparable in both products: 0.4–32.9 (9.3 ± 9.9) mm for MIM and 0.5–38.6 (11.0 ± 10.0) mm for Velocity, and correlated with the tumor diameter for MIM (*r* = 0.69, *P* = 0.002) and for Velocity (*r* = 0.68, *P* = 0.0003). Regarding the enhancement effect, the fiducial registration error was 1.0–24.9 (7.4 ± 7.7) mm for MIM and 0.3–29.6 (8.9 ± 7.2) mm for Velocity, which is shorter than that of plain CT (*P* = 0.04, for both).

**Conclusions:**

The DIR performance of both MIM and Velocity is comparable with regard to the liver. The fiducial registration error of DIR depends on the tumor diameter. Furthermore, contrast-enhanced CT improves the accuracy of both MIM and Velocity.

**Institutional review board approval:**

H28-102; July 14, 2016 approved.

## Background

The various organs of the human body are often deformed by irradiation. Reirradiation is sometimes conducted to treat new lesions that might occur. Before reirradiation is performed, it is vital to confirm the region irradiated by previous radiotherapy to avoid excess irradiation to the normal tissue as this could cause severe adverse effects. Primary liver tumors tend to recur inside the liver after treatment, and metastasis from other organs also makes it highly possible that new lesions will occur inside the liver. A substantial number of patients, therefore, require reirradiation to treat recurrence of cancer in the liver [1].

Thus, image registration is a particularly important issue for treating patients with liver tumors, even though, since the advent of particle beam radiotherapy, irradiated lesions are now well controlled [2–5]. To conduct image registration, an important problem must be considered: after irradiation, the irradiated area including the tumor is shrunk and the nonirradiated area is shifted and sometimes enlarged or shrunk, thus causing remarkable deformation of the liver in most patients [6]. This issue can be addressed by using deformable image registration (DIR), and a number of software products are now on the market. The use of DIR for applications and assessment of previously delivered irradiation doses is clinically expected to protect the normal liver tissue from receiving harmfully large doses of irradiation [7].

Several DIR algorithms exist, and DIR can be broadly classified into two categories: (1) intensity-based methods, which use a variety of image intensity metrics such as the gray scale, and (2) feature-based methods, which use specific image features such as contours [8]. Transformation models include optical flow-based equations [9], the “Demons” equation [10], B-splines [11], and thin-plate splines [12]. In most registration algorithms, the balance between image similarity and accurate matching of the local features on the one hand and deformation smoothness on the other hand is crucial to accurately measure the deformation [13]. The technique for evaluating the spatial accuracy of DIR involves landmark tracking [14] or contour or structure comparisons [15].

In recent years, advanced software equipped with the function of DIR has been developed for research purposes, and some of them are also available for clinical use. MIM Maestro (MIM Software Inc., OH, USA) (MIM) and Velocity AI (Velocity Medical Solutions, GA, USA) (Velocity) are the two most widely used in Japan and worldwide 2 of the 3 most widely used commercially available software products that can perform DIR and assist radiotherapy planning. In MIM, the DIR algorithm is intensity-based, free form cubic spline interpolation with essentially unlimited degrees of freedom [13]. MIM can dramatically deform the image, while, in cases with little contrast, it might lead to unreasonable deformation of the image. In Velocity, a B-spline deformable model is used [13]. Velocity uses standardized image intensity and has a smoothing and regularization function derived from the B-spline method. Although both software products are relatively new, some reports on their features and differences have been already been published. In brief, these are that MIM has the advantage in terms of small fields but sometimes produces registration error because of image noise, and Velocity has the advantage in terms of large fields. So far, there is no strong evidence for which software is superior [8, 13, 16].

We examined the spatial accuracy of DIR of MIM and Velocity after irradiation in the preliminary stage of deformation of the dose distribution in reirradiated liver tumors.

## Methods

We retrospectively reviewed patients who had received proton beam therapy (PBT) at our institute. All the study procedures involving human participants were conducted in accordance with the ethical standards of the institutional research committee and with the 1964 Helsinki declaration and its later amendments or comparable ethical standards. All the treatments were discussed at inhospital conferences, and informed consent was obtained from all the individual participants included in the study. The study received institutional review board approval (H28-102). We selected those patients who had a metallic material such as a fiducial marker or surgical clip (herein called *metallic marker*) already implanted very close to the liver tumor before PBT. We examined 24 consecutive patients treated between 2009 and 2014 (20 men, 4 women; aged 52–84 years). The most common disease was hepatocellular carcinoma (18 patients), followed by liver metastasis (5 patients) and intrahepatic bile duct carcinoma (1 patient). Fiducial markers for previous PBT were present in 21 patients, and surgical clips, in 3 patients. At our institute, abdominal computed tomography (CT) for diagnosis is usually not taken after metallic marker implantation, so these 21 patients had come to our hospital to receive PBT for new lesions in the liver. Twenty-two patients underwent irradiation for single lesions, and 2 patients, for 2 lesions. The total tumor diameter was 10-69 (median: 35) mm. The tumor was located in the left lobe in 5 patients, in the right lobe in 15 patients, and in both lobes in 4 patients. The distance between the tumor and the metallic marker was 5-33.7 (median: 12.0) mm. The total irradiation dose was 50-74 GyE in 22-37 fractions (Table 1).

**Table 1:** Characteristic of the patients

			Dislocation									
Number	Disease	materials	MIM (plain)	MIM (enhance)	Velocity (plain)	Velocity (enhance)	Tumor location	Tumor diameter	tumor-material distance	Dose (GyE/fr)	Duration (months)	Remark
1	HCC	marker	0.40	1.00	0.50	1.00	S8	35	11.5	72.6/22	9.4	
2	IHBDC	clip	3.00	2.30	18.00	14.30	S3	45	14.2	74/37	14.2	
3	meta	marker	6.70	8.70	9.20	7.70	S7	30	16.1	72.6/22	10.9	
4	HCC	clip	4.70	2.10	4.00	3.10	S4	20	5.2	72.6/22	10.3	
5	HCC	marker	1.80	2.10	4.60	0.30	S4/8	30	7.2	50/25	5.5	
6	meta	marker	15.20	8.20	20.50	4.50	S8	60	8.7	72.6/22	7.2	at
7	HCC	marker	8.20	5.40	22.30	20.40	S4/8	50	10.8	72.6/22	6.2	pl
8	meta	marker	1.30	1.60	4.00	2.20	S6	25	13.2	60/30	10.0	
9	HCC	marker	4.70	4.70	4.80	7.30	S7	25	5.2	50/25	10.9	
10	HCC	marker	1.80	2.20	1.90	2.00	S4	24	5.4	72.6/22	7.8	
11	HCC	marker	0.40	1.20	6.30	9.7	S5	35	5.7	50/25	12.1	
12	HCC	marker	18.10	19.10	4.70	16.60	S7	56	8.3	72.6/22	8.9	as
13	HCC	marker	22.90	5.90	18.30	12.50	S8	30	9.5	50/25	11.2	as
14	meta	marker	11.10	14.60	5.50	5.20	S4/8	47	9.6	60/30	7.2	pl
15	HCC	marker	32.90	18.90	21.40	13.80	S8	60	10.3	72.6/22	11.9	as
16	HCC	marker	11.30	2.30	10.70	7.70	S7	15	12.4	60/30	10.3	
17	HCC	marker	2.10	3.10	1.30	2.60	S1/4	35	14.9	60/30	8.0	as
18	HCC	marker	15.50	20.00	12.50	10.80	S7/8	38	16.1	72.6/22	6.5	
19	HCC	marker	0.80	1.20	3.40	3.10	S5/8	35	16.5	74/37	10.1	at
20	HCC	marker	0.70	2.00	9.70	9.00	S8	10	19.1	60/15	6.8	
21	HCC	marker	2.60	2.90	6.80	6.30	S1	24	30.9	72.6/22	8.7	
22	HCC	marker	29.60	24.90	38.60	S5/8	68	68	32.8	52.8/16	4.2	as
23	HCC	marker	24.60	20.80	30.70	19.10	S6,S8	69	33.0	70/35,66/10	12.7	as
24	meta	clip	3.00	2.50	3.20	4.60	S1,S5/8	40	33.7	45/15,72.6/22	13.4	

Abbreviations: *HCC* hepatocellular carcinoma, *IHBDC* intrahepatic bile duct carcinoma, *GyE/fr* Gray equivaent/fractions, *at*; atelectasis appearance during follow-up, *pl* pleural effusion appearance during follow-up, as ascites appearance or increased during follow-up

Duration means the interval to the post treatment CT. All unit of length is mm

Contrast-enhanced CT with the breath-holding technique was taken before and after treatment. The duration between PBT and posttreatment CT was 4-14 (median: 10) months. CT with a matrix resolution of 512*512 and a slice thickness of 5 mm was used. We used both plain and contrast-enhanced CT in our image analysis. For the patients who had dynamic contrast-enhanced CT, we used portal venous-phase CT. We performed rigid image registration (RIR) and then DIR of the pretreatment CT images. During the registration process, the priority area of calculation was manually designated to cover the whole liver. The fiducial registration error was assessed by examining the dislocation of the metallic marker from its position in the posttreatment CT and that in the deformed pretreatment CT images. We used the point at which the metallic density was the highest as the position of the metallic marker. The same process was performed using MIM (version 6.5.2) and Velocity (version 3.1.0).

The fiducial registration errors of MIM and Velocity were compared using both plain and contrast-enhanced CT and a paired *t* test. The Pearson product moment correlation was performed to examine the correlation between the fiducial registration error of RIR and DIR. Simple linear regression analysis was performed to examine the correlation between tumor diameter and fiducial registration error. Probability values below 0.05 were considered significant.

## Results

**Fig. 1:**
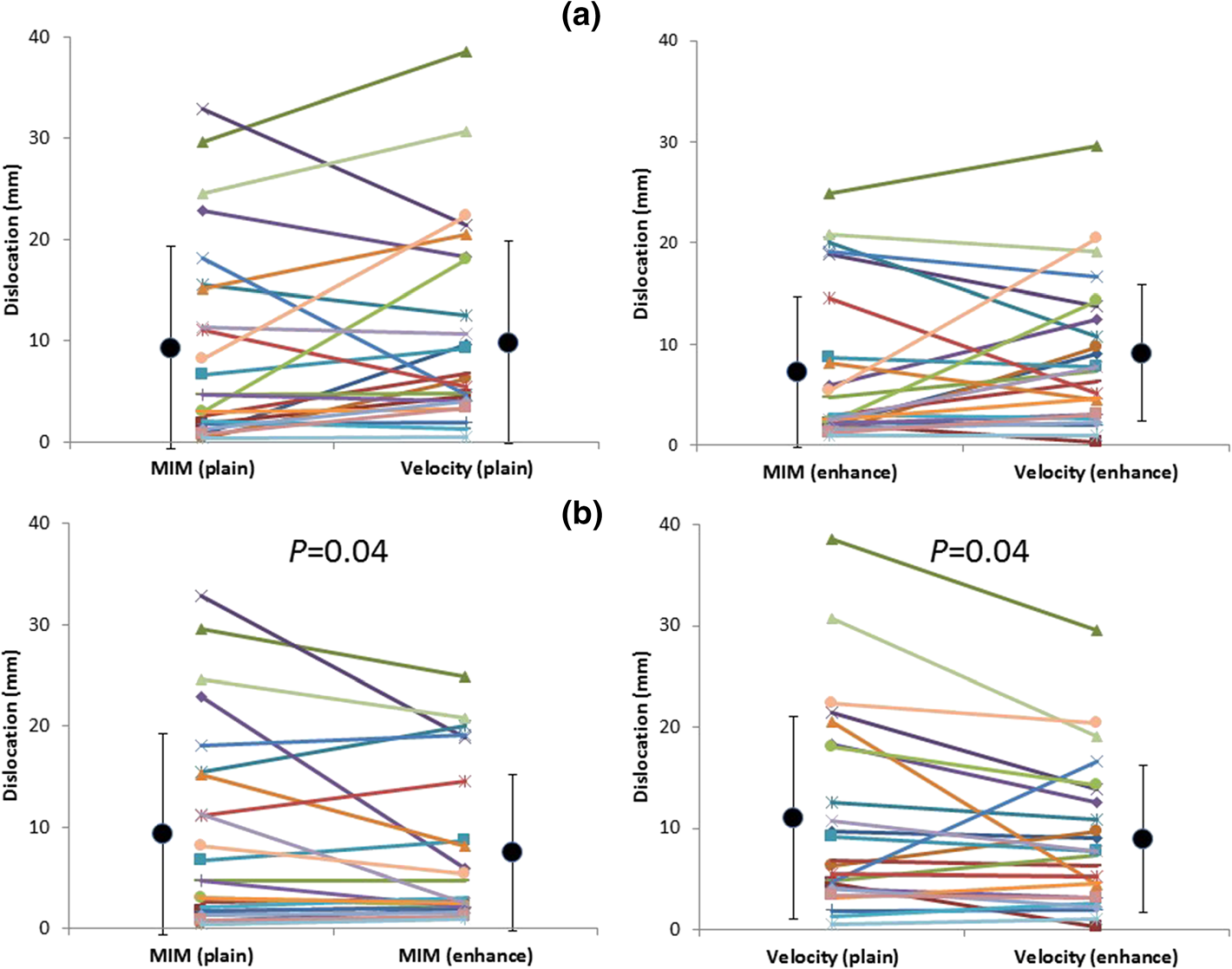
Fiducial registration error. **a** MIM and Velocity. Left: plain CT; Right: contrast-enhanced CT. **b** Plain and contrast-enhanced CT. Left: MIM; Right: Velocity

In the plain CT, the fiducial registration error was 0.4–32.9 (9.3 ± 9.9) mm for MIM and 0.5–38.6 (11.0 ± 10.0) mm for Velocity. The fiducial registration error was less for MIM in 16 patients and for Velocity in 8 patients; overall however, the results for both MIM and Velocity were similar (*P* = 0.18). In the contrast-enhanced CT, the fiducial registration errors for MIM (1.0–24.9 [7.4 ± 7.7] mm) and Velocity (0.3–29.6 [8.9 ± 7.1] mm) were also similar (*P* = 0.22) (Fig. 1a). As for the enhancement effect, the fiducial registration errors for MIM and Velocity were significantly shorter than they were in the plain CT (*P* = 0.04, for both). (Fig. 1b).

**Fig. 2:**
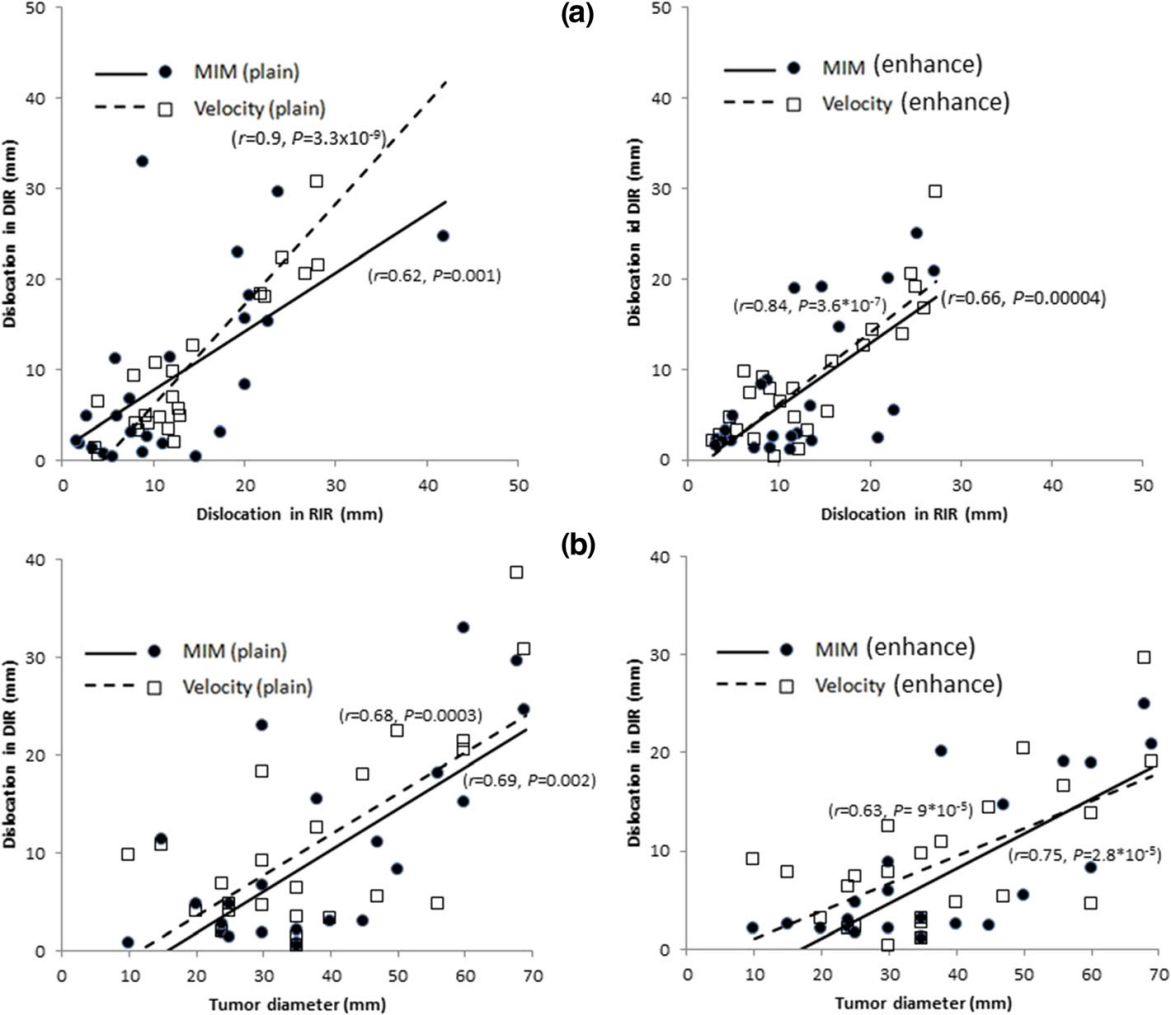
Correlation of the fiducial registration error. **a** RIR and DIR. Left: plain CT; Right: contrast-enhanced CT. **b** Tumor diameter and DIR. Left: plain CT; Right: contrast-enhanced CT

With regard to the fiducial registration error, DIR was significantly correlated with RIR for both MIM (*r* = 0.62, *P* = 0.001) and Velocity (*r* = 0.9, *P* = 3.3×10^-9^) in the plain CT (Fig. 2a). In the contrast-enhanced CT, DIR was also significantly correlated with RIR for both MIM (*r* =0.66, *P* = 0.0004) and Velocity (*r* = 0.84, *P* = 3.6 × 10^-7^). The fiducial registration error was significantly correlated with the tumor diameter for both MIM (*r* = 0.69, *P* = 0.002) and Velocity (*r* =0.68, *P* =0.0003) in the plain CT. In the contrast-enhanced CT, the fiducial registration error was also significantly correlated with the tumor diameter for both MIM (*r* =0.75, *P* = 2.8 × 10^-5^) and Velocity (*r* =0.63, *P* = 9 × 10^-5^). The tumor diameter predicted to produce a 10-mm fiducial registration error was 39.4 mm for MIM and 35.5 mm for Velocity in the plain CT and 45.6 mm for MIM and 42 mm for Velocity in the contrast-enhanced CT (Fig. 2b).

The tumor-marker distance did not differ according to the classification of the tumor location. However, cases in which the tumor in the right lobe had a trend toward the tumor-marker distance became large (Table 2).

**Table 2:** Cases with large tumor marker distance

MIM (plain)			MIM (enhance)			Velocity (palin)			Velocity (enhance)		
T. M. D. (mm)	T. L.	T. D. (mm)	Dis. (mm)	Loc.	T. D. (mm)	Dis. (mm)	Loc.	T. D. (mm)	Dis. (mm)	Loc.	T. D. (mm)
22.9	S8	30	20.0	S7/8	38	21.4	S8	60	20.4	S4/8	50
24.6	S6,S8	69	20.8	S6, S8	69	22.3	S4/8	50	29.6	S5/8	68
29.6	S5/8	68	24.9	S5/8	68	30.7	S6, S8	69			
32.9	S8	60				38.6	S5/8	68	68		

Abbreviations: *T. M. D.* tumor marker distance, *T. L* tumor location, *T. D* tumor diameter

**Fig. 3:**
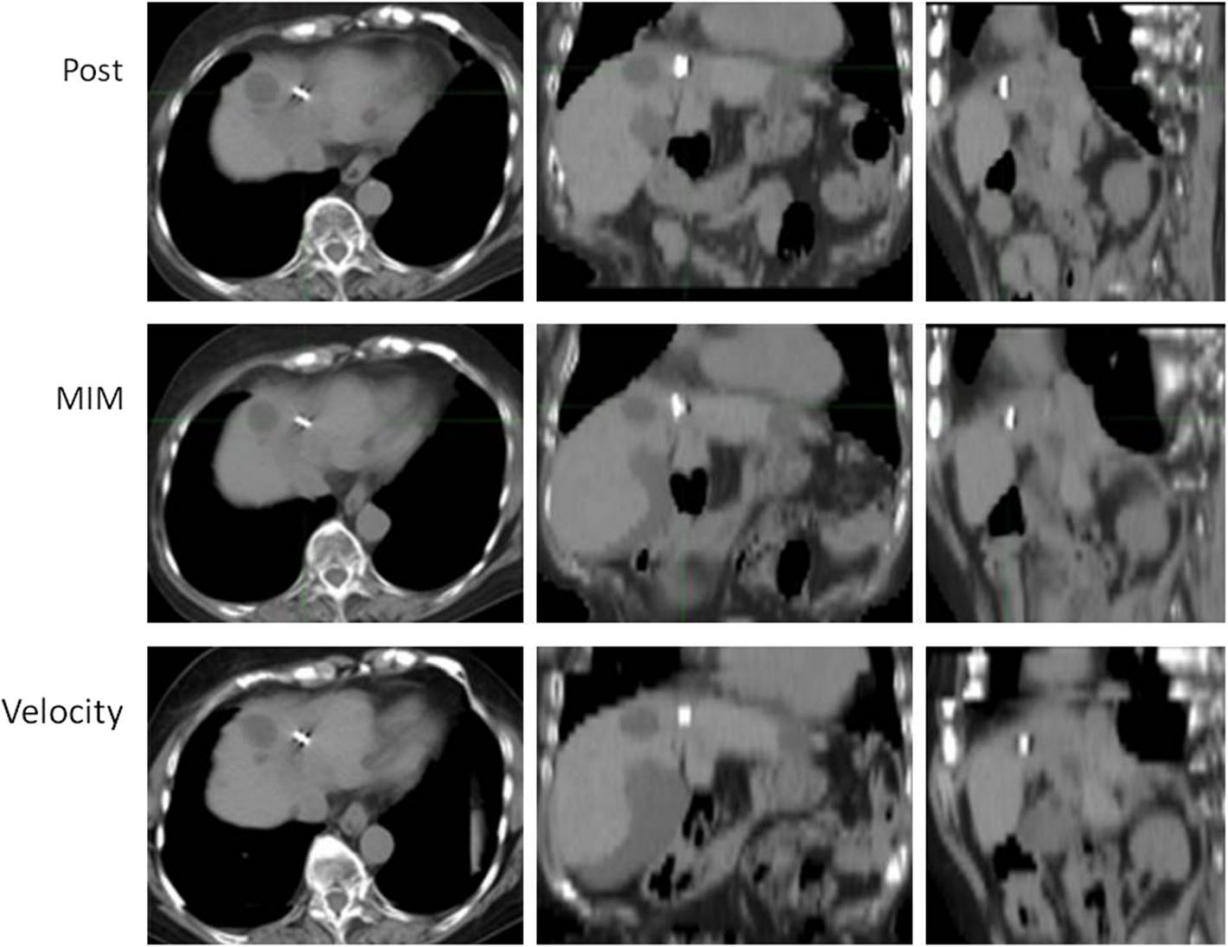
A case with a small registration error for both MIM and Velocity. Proton beams at 72.6 GyE were delivered to the tumor in S4 7.8 months before. The fiducial registration error was 1.8 mm in MIM and 1.9 mm in Velocity, the shortest among all the patients

**Fig. 4:**
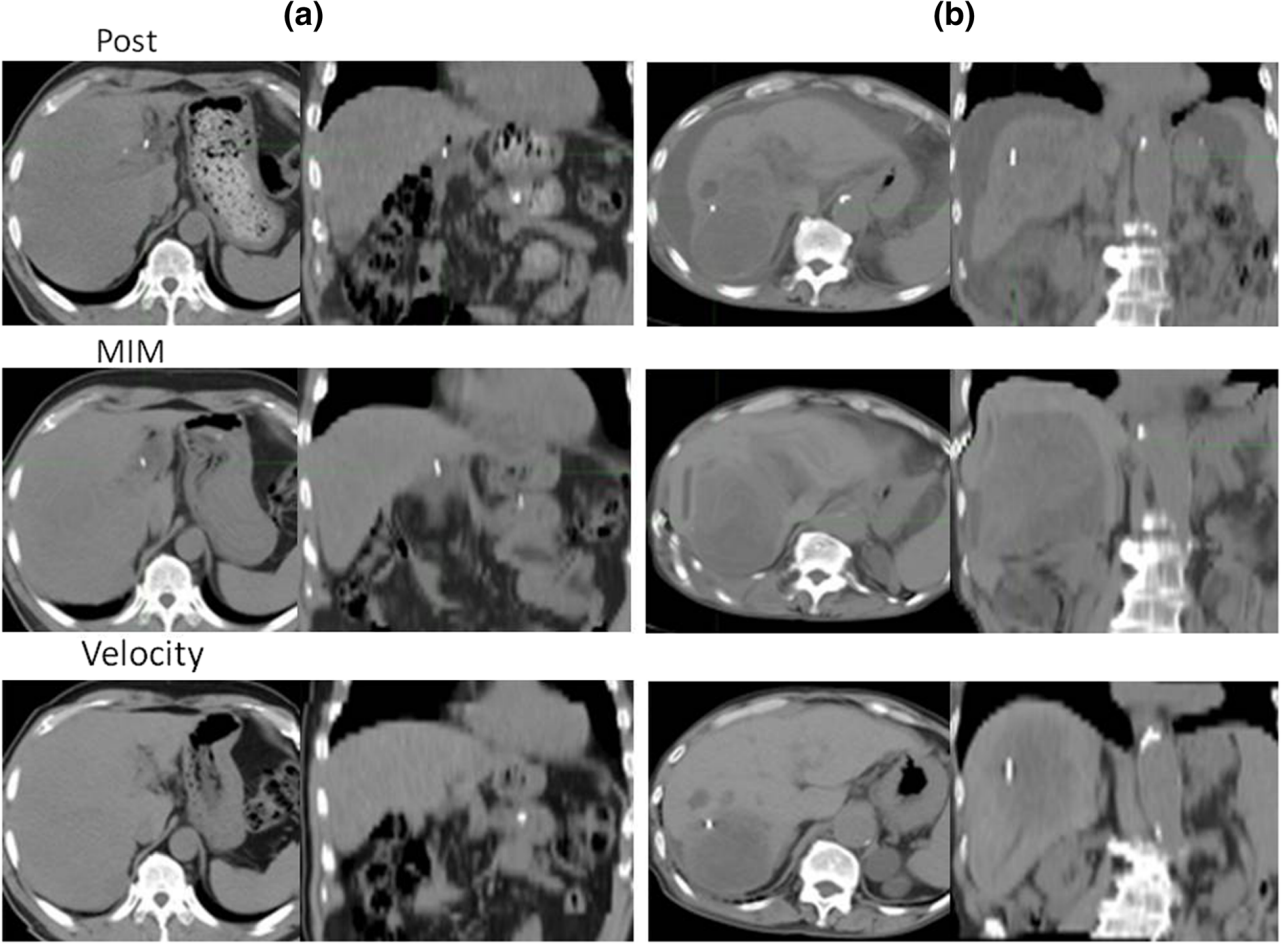
Cases with large discrepancies in the registration error between MIM and Velocity. **a** Proton beams at 74 GyE were delivered to the tumor in S3 14.2 months before. The fiducial registration error was 3 mm in MIM and 18 mm in Velocity, the largest discrepancy (Velocity-MIM) among all the patients. **b** Proton beams at 72.6 GyE were delivered to the tumor in S7 8.9 months before. The fiducial registration error was 18.1 mm in MIM and 4.7 mm in Velocity, the largest discrepancy (MIM-Velocity) among all the patients

**Fig. 5:**
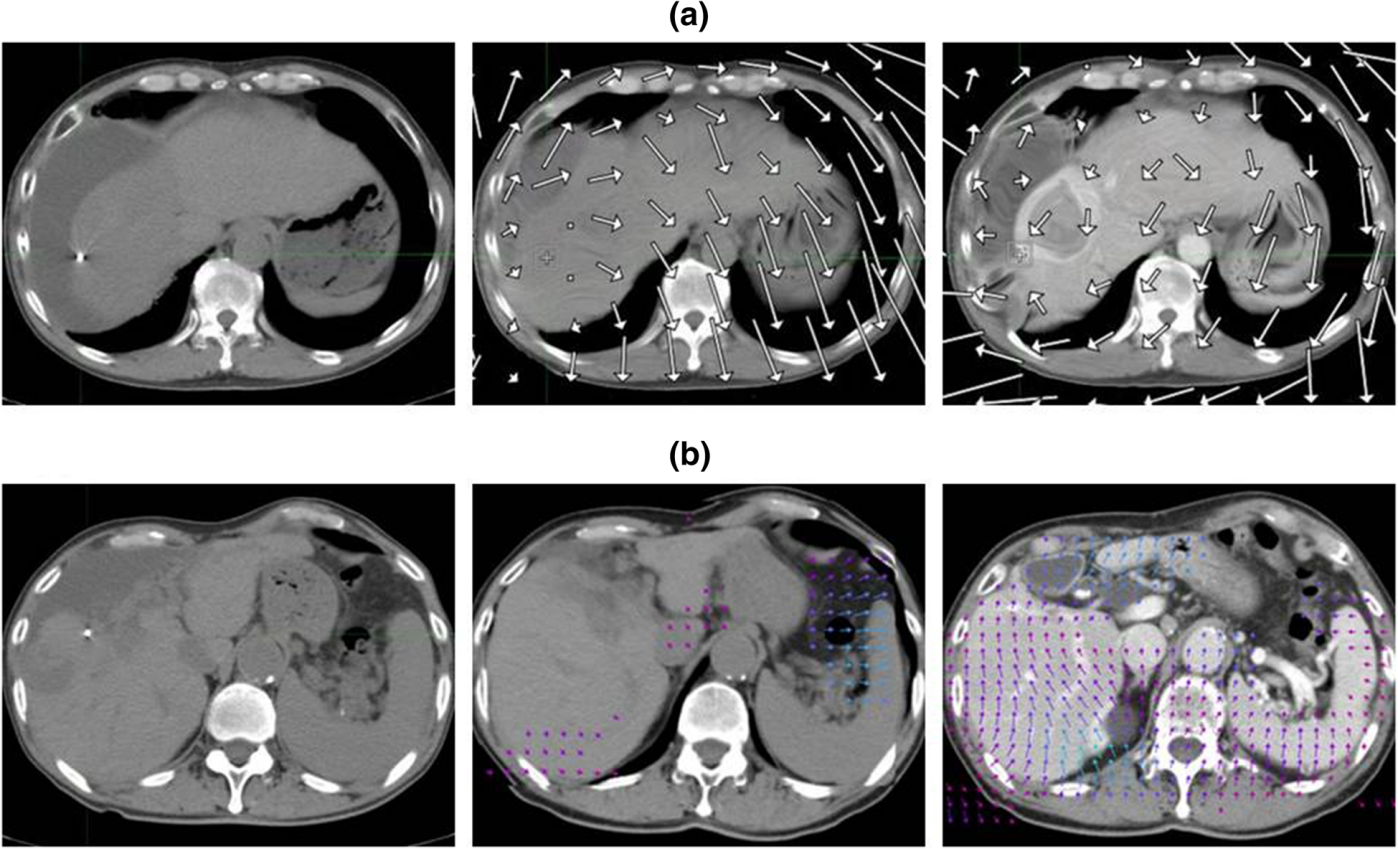
Cases with large discrepancies in the deformation pattern between plain and contrast-enhanced CT. **a** Proton beams at doses of 50 GyE were delivered to the tumor in S8 11.2 months before. Left: posttreatment CT; middle: deformed plain CT; right: deformed contrast-enhanced CT. The vector expressed by MIM means the fiducial registration error in each area. **b** Proton beams at doses of 70 and 66 GyE were delivered to the tumor in S6 and S8 12.7 months before. The vector expressed by Velocity means the fiducial registration error in each area

Figure 3 shows a case in which the fiducial registration error was small and similar for both MIM and Velocity. Figure 4 shows the cases that showed the biggest discrepancies between MIM and Velocity in terms of the fiducial registration error. Figure 5 shows the cases in which the DIR results were greatly affected by the contrast enhancement effect. All the deformed images showed slice levels that corresponded to the location of the metallic marker on the posttreatment CT.

## Discussion

We compared the capabilities of MIM and Velocity. At first, we considered the advantages of MIM. Although the fiducial registration error of DIR correlated with that of RIR both for MIM and for Velocity, there were some differences in the degree of fiducial registration error. As shown in Fig. 2(a), the correlation coefficient for Velocity was 0.9, which is an extremely strong correlation, whereas for MIM it was 0.62, which is only a moderate correlation. Figure 4(a) shows the case with the largest difference in fiducial registration error between Velocity and MIM. The metallic marker could not be found in the axial, coronal, or sagittal image in the RIR process of both MIM and Velocity. However, during the DIR process, MIM could correctly identify the location in which the metallic marker was observed in any of the directional images. In contrast, the DIR process of Velocity shifted the location to a position in which the metallic marker was not observed in any of the directional images. Yeo et al reported that the magnitude of deformation has a much larger effect on the accuracy of registration than does the complexity of deformation [17]. Our results suggest that the DIR process in Velocity seems to be more dependent on the RIR, while MIM has a greater ability to modify during the process of DIR. Next, we considered the advantages of Velocity. As shown in Fig. 4(b) of an irradiated tumor in the right lobe, MIM showed an unnaturally deformed low-density tumor, especially in the coronal image, whereas Velocity showed the natural shape of the tumor. Previous studies have shown that MIM produces beautiful image similarity but may produce nonphysical deformation fields, while Velocity produces smooth, physically plausible deformation fields [18]. It seems that MIM has higher flexibility and causes excellent overall spatial accuracy; although it tends to deform forcibly. This could sometimes cause unreasonable DIR and diminish the ability to transfer contours.

As shown in Fig. 1(a), although the fiducial registration errors of MIM and Velocity were similar overall, they were not necessary closely correlated, and for some patients either MIM or Velocity had a distinct advantage. Some previous studies reported that it is hard to decide which software is impartially superior to others in terms of DIR accuracy [8, 13, 18]. Our results also demonstrate that the superiority of DIR accuracy for the liver varies by patient, making it difficult to state which software is better. It is generally understood that DIR will work well with feature-rich images in which there is little or no ambiguity between corresponding points in the source and target images. The liver is one of the organs that have a relatively homogeneous Hounsfield unit (HU) and a lack of morphological characteristics. Therefore, one important objective of this study was to determine how well these software products can perform DIR in low-contrast organs such as the liver. We analyzed the portal vein phase image, which enhances a greater number of vessels. As we expected, contrast-enhanced CT could accomplish on average 1.9-mm less fiducial registration error than could plain CT in MIM. Moreover, Velocity could also, on average, accomplish a 2.1-mm enhancement effect, as shown in Fig 1(b). In addition, the enhancement effect could change the deformation pattern. As shown in Fig. 5, the vector went toward the right posterior direction in the plain CT; by contrast, the vector circled in a clockwise direction in the contrast-enhanced CT of MIM. Similarly, in the plain CT, the vector moved slightly backwards only in the peripheral region of the liver, whereas in the contrast-enhanced CT of Velocity, the vector moved forward through most of the regions of the liver. We are convinced that in the DIR of both MIM and Velocity, enhancement-derived contrast not only works toward spatial accuracy but also changes the deformation pattern, such as the linear and rotational directions.

We used a metallic marker to calculate the image registration accuracy because it is difficult to measure the registration error of the tumor itself and because precise contouring of the whole liver by distinguishing the liver from the porta hepatis is not completely reproducible in each study. It is feasible to calculate the fiducial registration error at multiple points in each patient. However, in the daily clinical setting, the number of implanted metallic markers is usually 1 or 2. Therefore, we selected only the patients whose metallic materials were close to the tumor. In this study, the metallic markers were implanted close to the tumor, with a range of 5–33.7 (median: 12.0) mm. Thus, we consider the metallic markers to act as surrogates of the fiducial registration error in DIR. There may be criticism that the high HU of the metallic markers could have affected the image registration. However, in all the patients, the metallic artifact was so small when compared with the large volume of the liver, and therefore, we think that the accuracy of the image registration is seldom affected by the artifact. Moreover, we routinely compare and conduct image registration of CTs with tiny metallic markers already implanted. Therefore, this analysis reflects the conditions of the daily clinical setting, and we consider that analysis using CT with implantation of tiny metallic markers is clinically allowable.

Both MIM and Velocity have rapidly expanded the market for such types of software, and new software products have been manufactured or are planned for manufacture to meet the demand for high-precision radiotherapy. It is expected that several types of commercial-based software products equipped with the DIR function will be developed, not only for examination of previous radiotherapy planning, but also for use in adaptive radiotherapy. In this study, we investigated the registration error by using clinical data. However, it is also important to investigate and validate the registration error by using phantoms. We are considering a phantom study as the next step to prove the registration error that we concluded in this clinical study.

## Conclusion

For image registration of the liver, the DIR performances of MIM and Velocity are similar overall. However, which software is the better option varies according to the patient. The spatial accuracy of DIR depends on the accuracy of RIR and also on the tumor diameter. Finally, contrast-enhanced CT improves the accuracy of both MIM and Velocity.

## Abbreviations

CT: (Computed tomography): image used by computer-processed combinations of many X-ray images
DIR: (Deformable image registration): image registration technique with deformation
HU: (Hounsfield unit): A quantity commonly used in CT to express CT numbers in a standardized and convenient form
MIM: (MIM Maestro): name of software
PBT: (Proton beam therapy): a type of radiotherapy using proton beams
RIR: (Rigid image registration): image registration technique horizontally and rotationally without deformation
Velocity: (Velocity AI): name of software
